# Supplementary material for: Pre-Clinical Evaluation of the Antiviral Activity of Epigalocatechin-3-Gallate, a Component of Green Tea, against Influenza A(H1N1)pdm Viruses
Source: Viruses. 2023 Dec 16;15(12):2447. doi: 10.3390/v15122447 (PMC10747412; doi:10.3390/v15122447)
Supplement: Supplementary file 1 [file viruses-15-02447-s001.zip › viruses-2712767-supplementary.pdf]

Supplementary Information

Supplementary Table S1. Ferret sera biochemistry at day 0, 4, and 14 post treatment onset of EGCG treatment for 5 days (d0-d4) with 125 mg/kg twice daily compared to reference ranges from Hein et al. [41].

Day	0						4						14						Ref. Range
Treatment	Placebo			EGCG			Placebo			EGCG			Placebo			EGCG			
Ferret	1	2	3	4	5	6	1	2	3	4	5	6	1	2	3	4	5	6	
**Enzymes (IU/L)**																			
ALT	228	85	183	431	333	167	236	82	261	534	239	132	244	69	221	378	251	106	49.0–242.8
AST	168	137	120	133	191	120	150	114	124	171	108	116	85	58	78	110	81	79	40.1-142.7
ALP	10	8	35	52	und	52	29	21	39	69	30	25	29	23	37	70	33	28	13.3–141.6
GGT	1	1	5	4	1	1	2	1	4	4	2	1	1	1	1	4	3	1	0.2–14.0
Amylase	34	und	43	35	38	31	39	25	29	42	37	29	29	22	40	38	34	34	19.4–61.9
Lipase	58	54	48	39	74	34	30	33	32	33	40	30	30	41	34	32	37	32	73.2–351.1
CK	446	252	324	211	472	321	417	244	276	295	250	261	151	114	232	229	201	250	94.0–730.9
**Substrates**																			
Bilirubin (µM/L)	<1	<1	<1	<1	<1	<1	<1	<1	<1	<1	<1	1	1	1	1	1	1	1	0.0–3.3
Albumin (g/L)	33	34	33	32	38	31	29	33	33	32	30	34	27	30	29	33	29	33	28.0–43.9
Protein (g/L)	77	63	60	63	76	58	64	63	58	64	60	63	58	57	53	62	56	59	54.7–77.9
Cholesterol (mM/L)	5	5.7	8.7	8	4.5	5	5.2	5	6.1	9.3	4.9	5.1	5.8	4.9	9.5	8.8	3.8	5.4	2.4–7.1
Triglycerides (mM/L)	0.7	0.8	1.1	1	0.5	1.1	0.5	0.5	0.5	1	0.5	0.8	0.4	0.7	0.8	1.1	0.3	0.4	0.5–2.8

**Glucose (mM/L)**	4.2	3.6	4.2	3.4	4.1	3.7	5.7	5.2	7	4.9	5.4	8.1	6.4	5.5	7	5.6	6.4	7.2	3.0–8.5
**Urea (mM/L)**	10	11	12	12	10	11	9	10	10	13	12	6	9	12	10	14	11	7	4.8–16.9
**Creatinine (μM/L)**	54	59	56	55	58	51	37	45	39	43	57	19	47	50	34	32	43	34	23.0–76.7
**Electrolytes (mM/L)**																			
**Sodium**	150	151	152	151	147	148	148	149	151	149	149	147	148	152	151	149	149	150	140.1–169.7
**Potassium**	5.1	5.5	4.8	5.3	5.6	5	4.8	5.2	4.8	5	5.2	5	5.4	5.7	4.8	4.9	5.5	5	1.0–3.1
**Chloride**	119	119	116	117	114	121	118	120	116	116	115	114	118	121	116	119	118	118	108.0–119.9
**Calcium**	1.9	2.0	2.1	2.1	2.0	2.1	2.1	2.1	2.2	2.2	2.2	2.1	2.0	2.1	2.2	2.2	2.1	2.2	2.0–2.6
**Phosphate**	1.7	1.8	2.0	1.9	2.1	1.9	1.6	1.6	2.3	2.0	2.0	1.8	1.9	2.2	2.3	2.5	2.2	1.7	1.0–3.1
**Magnesium**	1.0	1.0	0.9	0.9	1.0	0.9	1.0	1.0	0.9	1.0	1.0	1.0	0.9	1.0	0.9	0.9	0.9	0.9	0.9–1.6

ALT, Alanine transaminase; AST, Aspartate transaminase; ALP, Alkaline phosphatase; GGT, Gamma-glutamyl transferase; CK, Creatine kinase.

Supplementary Table S2. Ferret nasal wash and tissue virus genome pyrosequencing resulting proportion of NA-H275Y oseltamivir resistance mutation.

Treatment	Ferret	Nasal wash D4	Lung left cranial	Lung right cranial	Soft palate	Nasal turbinate
**Placebo**	**1**	<5%	<5%	5.8%	<5%	<5%
	**2**	<5%	<5%	<5%	<5%	<5%
	**3**	<5%	<5%	<5%	<5%	<5%
	**4**	<5%	<5%	<5%	<5%	<5%
**EGCG**	**5**	<5%	Undetected	Undetected	<5%	<5%
	**6**	<5%	<5%	<5%	<5%	<5%

	**7**	<5%	Undetected	<5%	Undetected	<5%
	**8**	<5%	<5%	<5%	<5%	<5%
**OST**	**9**	<5%	Undetected	Undetected	<5%	<5%
	**10**	<5%	<5%	<5%	Undetected	<5%
	**11**	<5%	<5%	<5%	8.4%	<5%
	**12**	<5%	<5%	Undetected	<5%	<5%
**EGCG + OST**	**13**	<5%	<5%	Undetected	<5%	<5%
	**14**	<5%	<5%	Undetected	<5%	<5%
	**15**	<5%	Undetected	Undetected	5.2%	<5%
	**16**	<5%	<5%	6.7%	<5%	<5%
